# Integration of sarcopenia screening into radiotherapy planning: Validation of a time‐efficient SMI measurement method using MIM software in prostate cancer

**DOI:** 10.1002/acm2.70694

**Published:** 2026-07-09

**Authors:** Pauline De Bruyn, Nicolas Coquelet, Paulo Ferreira, Madeline Michel, Thomas Descamps, Robbe Van den Begin, Dirk Van Gestel, Jean‐Charles Preiser

**Affiliations:** ^1^ Department of Radiation Oncology Université libre de Bruxelles Hôpital Universitaire de Bruxelles Institut Jules Bordet Brussels Belgium; ^2^ Department of Radiology Université libre de Bruxelles Hôpital Universitaire de Bruxelles Institut Jules Bordet Brussels Belgium; ^3^ Université libre de Bruxelles Brussels Belgium; ^4^ Department of Internal Medicine Université libre de Bruxelles Hôpital Universitaire de Bruxelles Institut Jules Bordet Brussels Belgium

**Keywords:** automated workflow, MIM‐software, muscle segmentation, prostate cancer, radiotherapy, sarcopenia screening, skeletal muscle index (SMI)

## Abstract

**Background:**

Sarcopenia (SP) affects 43.8% of men with prostate cancer (PCa), correlating with higher all‐cause mortality and reduced quality of life. Systematic SP screening could reduce treatment complications and improve prognosis. Although several validated software tools exist for CT‐based skeletal muscle assessment, their implementation in routine clinical practice remains limited due to time constraints, operator dependency, and lack of integration into radiotherapy planning systems.

**Purpose:**

We aimed to validate a new SP screening method using MIM® software, a program commonly used in radiotherapy planning, to facilitate its implementation in daily practice.

**Methods:**

In 41 patients with PCa, Skeletal Muscle Index (SMI) was retrospectively calculated using MIM® and compared with the validated ImageJ® method. Agreement was assessed via Bland‐Altman analysis, while sarcopenic status was classified using literature‐based SMI thresholds. Measurement time and reproducibility were evaluated using Wilcoxon tests, Intraclass Correlation Coefficient (ICC), and Fleiss kappa coefficients (κ). Additionally, an automated MIM‐workflow was developed and compared to the semi‐manual MIM‐method in terms of agreement and processing time.

**Results:**

The cohort had a median age of 71 years, with 66% overweight or obese and 22% classified as sarcopenic. The new method demonstrated high concordance with the validated method (mean SMI difference: −0.04 cm^2^/m^2^, *p* = 0.23), while significantly reducing calculation time (3.5 ± 1 min vs. 10 ± 1 min, *p* < 0.001). Reproducibility was excellent, with an ICC of 0.99 for SMI and perfect agreement on sarcopenic classification (*κ* = 1). The automated MIM‐workflow further decreased processing time (median 3.2 min vs. 3.5 min, *p* < 0.001) but yielded slightly higher SMI values (mean difference: 2.3 cm^2^/m^2^, *p* < 0.0001).

**Conclusion:**

MIM® software provides a reproducible, reliable, and time‐efficient alternative for SP screening, allowing radiation‐oncologists to implement this assessment immediately into routine radiotherapy planning. Although the automated MIM‐workflow requires further calibration to correct for systematic SMI overestimation, it represents a promising step toward full automation and broader clinical integration of SP assessment within radiation‐oncology.

## 1 INTRODUCTION

In men with Prostate Cancer (PCa), sarcopenia (SP) has a high prevalence of 43.8%, is associated with increased all‐cause mortality^1^ and with a worse health‐related quality of life in advanced PCa patients.^2^

The management of SP and its impact on treatment‐related complications are gaps that still need to be addressed.^3^ Current evidence therefore highlights the need for systematic SP assessment in clinical practice.^1^, ^4^

The most recent definition of SP proposed by the European Working Group on Sarcopenia in Older People (EWGSOP) includes a reduction in muscle mass as a key component. In oncology, assessment of muscle mass using Computed‐Tomography (CT) scanners has become widespread, as CT imaging is routinely performed for diagnosis, staging, and follow‐up. Muscles delineation is commonly performed on CT slices at the level of the third lumbar vertebra (L3)^5^ enabling calculation of the Skeletal Muscle Index (SMI). This index can then be compared to sex‐ and Body Mass Index (BMI)‐specific cut‐off values,^6^ to determine the presence or absence of SP.

## 2 THEORETICAL FRAMEWORK

Several software programs have been validated for CT‐based skeletal muscle quantification, including commercial tools such as SliceOmatic®, radiology viewers such as OsiriX®, and open‐source platforms such as ImageJ® and 3D Slicer. A comparative overview of the main software tools, including their cost, level of automation, and key validation references, is presented in Table 1.

**TABLE 1:** Comparison of software tools for CT‐based skeletal muscle analysis.

Software	Cost^a^ (USD, indicative)	Segmentation approach	Integration into clinical workflow^b^	Key validation references
SliceOmatic®	Commercial (∼$4,000, perpetual license)	Semi‐automatic (HU‐based thresholding + manual correction)	No	Rollins 2019^7^; van Vugt 2017^8^; Prado 2008^9^
OsiriX® / Horos®	Commercial (∼$699/year, OsiriX MD) / Free (Horos)	Semi‐automatic (ROI‐based + HU thresholding)	Partial^c^	Rollins 2019^7^; Barbalho 2019^10^; van Vugt 2017^8^; Reisinger 2015^11^
ImageJ® / Fiji®	Free (open‐source)	Manual or semi‐automatic (plugin‐based HU thresholding)	No	Irving 2007^12^; Fortin 2012^13^; van Vugt 2017^8^; Teigen 2018^14^
3D Slicer® / BodyCompSlicer	Free (open‐source)	Semi‐automatic (extension‐based; optional AI modules)	No	Viddeleer 2024^15^; Takahashi 2017^16^
Mimics®	Commercial (∼$10,000–$20,000+, variable license)	Semi‐automatic (advanced segmentation tools)	No	Viddeleer 2024^15^
CoreSlicer®	Free (web‐based)	Automatic (HU‐based thresholding)	No	Mullie 2019^17^; Jiménez‐Sánchez 2024^18^
SarcoMeas	Free (academic use)	Semi‐automatic (HU‐based segmentation)	No	Viddeleer 2024^15^
Aquarius iNtuition®	Commercial (enterprise pricing, ∼$5,000–$15,000/year)	Semi‐automatic (HU‐based segmentation)	Partial^c^	Viddeleer 2024^15^
FatSeg (MeVisLab)	Free (academic use)	Semi‐automatic (HU‐based segmentation)	No	Rollins 2019^7^; van Vugt 2017^8^
Comp2Comp (AI)	Free (open‐source)	Fully automatic (deep learning–based segmentation)	No	Blankemeier 2023^19^; Hofmann 2025^20^
TotalSegmentator	Free (open‐source)	Fully automatic (deep learning–based segmentation)	No	Wasserthal 2023^21^
TissueCompass	Free (research use)	Semi‐automatic (AI‐assisted segmentation)	No	Imani 2022^22^

^a^ Cost categories reflect licensing and access conditions. “Open‐source” indicates publicly available code, whereas “web‐based” tools are accessed through a browser and may involve external data processing.

^b^ Integration into clinical workflow refers to the ability to perform skeletal muscle analysis directly within software routinely used for patient management, without requiring data export or additional tools.

^c^ Partial integration refers to software integrated into clinical imaging workflows (e.g., PACS or radiology platforms) but not into radiotherapy planning systems.

As shown in Table 1, all commonly used software tools have been validated using various methodologies. Notably, although these tools differ in usability and level of automation, their measurement outputs are highly comparable, highlighting that current limitations are primarily related to implementation rather than measurement accuracy. Indeed, most approaches remain time‐consuming, operator‐dependent, or require the use of external software—sometimes associated with additional costs—and none are routinely integrated into radiation oncology workflow, which may limit their adoption in daily clinical practice.

Among these tools, ImageJ® is widely used due to its accessibility and the availability of standardized step‐by‐step protocols.^23^ It is therefore commonly considered a reference method for research purposes.

Radiation‐oncologists (ROs), who routinely use CT‐based planning software such as MIM®—implemented in more than 3,000 institutions worldwide^24^ ‐ for tumor and organs‐at‐risk delineation, represent a key group for integrating SP assessment into daily practice. Embedding SMI calculation directly into existing tools such as MIM® aligns with the premise that integrating validated clinical measures into established workflows improves feasibility, reproducibility, and ultimately patient care. Since radiotherapy (RT) is one of the standard treatments for PCa,^25^ enabling ROs to assess muscle mass using their existing RT planning software represents a valuable opportunity to implement SP screening in a vast population known to be at risk.

Grounded in this framework, the present study evaluates whether SMI measurement using MIM® provides accuracy comparable to the validated method (ImageJ®), while enhancing time efficiency and clinical feasibility.

## 3 METHOD

We retrospectively collected pelvic CT scans of 41 consecutive PCa patients undergoing preparation for RT. All CTs were acquired using the Siemens® spiral CT scanner of the RT department at the Institut Jules Bordet. CT images were either contrast‐enhanced or unenhanced with 2 or 3‐mm slice thickness. We collected patients’ height and weight for body surface area and BMI calculation. On each CT, we selected two non‐adjacent axial slices from the same series, at the level of the L3.

ImageJ® was selected as the reference method due to its widespread use in the literature and availability of standardized protocols. We imported the selected slices and opened them in the previously downloaded ImageJ® software to delineate the muscles following the step‐by‐step guide of Gomez‐Perez et al.^23^ Based on the measured Skeletal Muscle Volume (SMV), we calculated the Skeletal Muscle Area (SMA) using the blank preprogrammed Excel template provided on the ImageJ website. The mean of the two SMA values was then divided by the body surface area to obtain the SMI.

**FIGURE A1:**
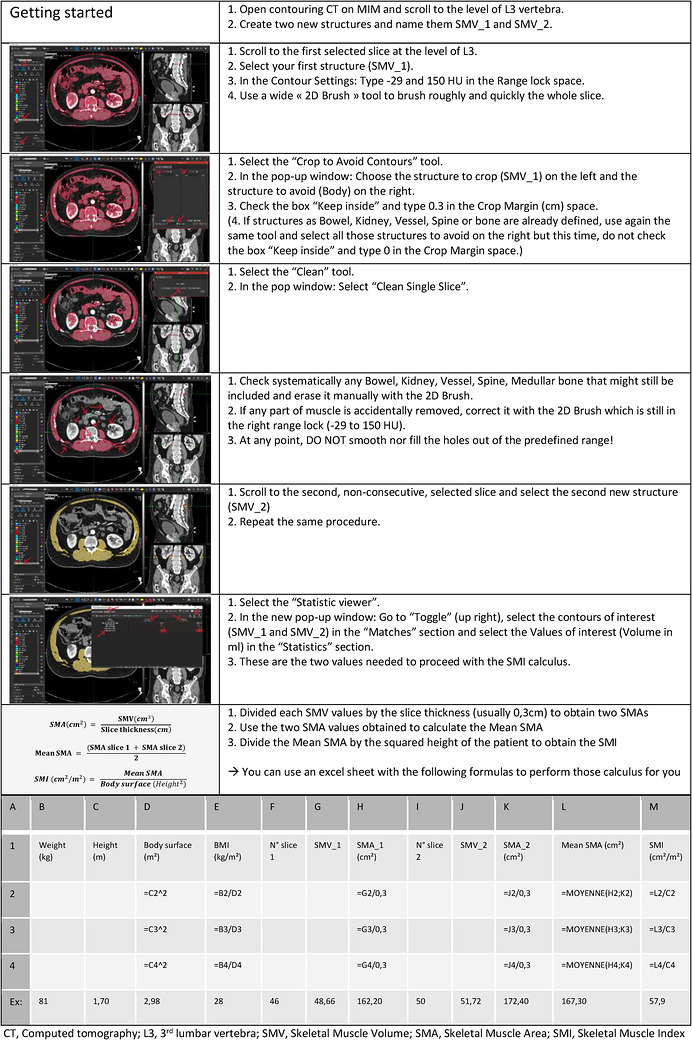
Full step‐by‐step guide and Excel of the MIM‐method. Illustration of the step‐by‐step guide to measure the Skeletal Muscle Volume (SMV) on CT‐slice with the MIM® software and to calculate the Skeletal Muscle Index (SMI) with the Excel sheet template.

In parallel, the same slices were processed with MIM® software to delineate muscles within a Hounsfield Unit (HU) range of −29 to +150 HU, yielding the SMV for each slice. These values were entered into a custom‐designed Excel sheet to calculate the corresponding SMI (Full step‐by‐step guide and Excel in Appendix; Figure A1; Formulas in Appendix; Table A1).

This approach is based on standard Hounsfield unit–based muscle segmentation, but differs from conventional methods by embedding SMI calculation directly within a radiotherapy planning software routinely used in clinical practice.

Those two approaches for obtaining SMI values will hereafter be referred to as the “ImageJ‐method” and the “MIM‐method”, respectively. To compare the SMI values derived from each method, we computed a Bland‐Altman plot and recorded the time required from the first CT image opening to the final SMI calculation for both methods.

We used paired *t*‐tests (or Wilcoxon signed‐rank test, as appropriate) to assess differences in SMI and computation time. A two‐tailed *p* < 0.05 was considered statistically significant.

Based on the sex‐specific and BMI‐adjusted SMI cutoffs proposed by Martin et al.^6^ and Gomez‐Perez et al.^23^, patients were classified as sarcopenic or non‐sarcopenic. Men with a BMI ≥ 25 kg/m^2^ were considered sarcopenic if their SMI was < 53cm^2^/m^2^ while men with a BMI < 25 kg/m^2^ were considered sarcopenic if their SMI was < 43cm^2^/m^2^. We then assessed whether the sarcopenic classification differed according to the method used to calculate the SMI.

**FIGURE A2:**
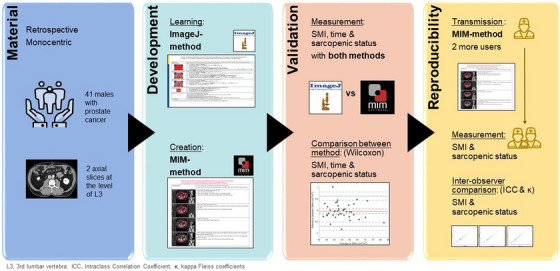
Illustration of the full MIM‐method validation's study design. Illustration of the method showing the material used, the development of learning skills and of the new method using MIM®, the validation of the new method by comparing it to a previously validated method (using ImageJ software) and the reproducibility assessment of the new MIM‐method by comparing the results of three independent users.

To evaluate inter‐investigator reproducibility, a step‐by‐step guide describing the MIM‐method was provided to two additional investigators, who independently calculated SMIs and determined sarcopenic status for the same patient cohort. Both investigators were blinded to the first investigator's results and to each other's data. We used an Intraclass Correlation Coefficient (ICC) and a kappa Fleiss coefficient (k) to investigate the inter‐investigator variability in SMI measurement and sarcopenic status assessment (Full method illustration in Appendix; Figure A2). Additionally, a qualitative interview was conducted with the two independent investigators to assess the perceived burden of the MIM‐method.

To further support ROs, an automated workflow of the “MIM‐method” was developed using MIM® Workflow Builder, aiming to streamline and standardize SP assessment. The workflow begins with a guided manual input of patient height and weight, and manual contouring of one sagittal and coronal slice on the 3^rd^ lumbar vertebra (L3) to serve as a reference. It then automatically contours the entire body using optimized CT window settings, isolates the L3 region, and performs muscle segmentation. After optional manual corrections, the workflow calculates and exports key metrics, including BMI, SMV, SMA, Body Surface Area, and SMI. (Detailed automated MIM‐workflow building guide in Supporting Information).

A Bland‐Altman plot was also generated to compare SMI values obtained using the automated workflow with those derived from the semi‐manual MIM‐method. Computation time were recorded, and paired *t*‐tests (or Wilcoxon signed‐rank test, as appropriate) were again used to assess differences in SMI and time, with statistical significance set at *p* < 0.05 (two‐tailed).

## 4 RESULTS

The MIM‐method proved to be reliable, time‐efficient, and reproducible for CT‐based SP assessment.

The cohort consisted of 41 male patients with PCa, with a median age of 71 years (range: 56–86). The majority were overweight or obese (42% overweight, 24% obese). We identified SP in 22% of patients, all of whom were either overweight (78%) or obese (22%) (Population characteristics; Table 2).

**TABLE 2:** Population characteristics.

DEMOGRAPHIC DATA	
N	41
Age ^a^, *year*	71 (56–86)
BMI ^b^	
Normal [18,5 – 24.9]	14 (34)
Overweight [25 – 29.9]	17 (42)
Obese [30 – 40]	10 (24)
Sarcopenic status ^b^	
Sarcopenic	9 (22)
Non‐sarcopenic	32 (78)

Abbreviation: BMI: Body Mass Index.

^a^ Median (min‐max).

^b^ *n* (%).

**FIGURE 1:**
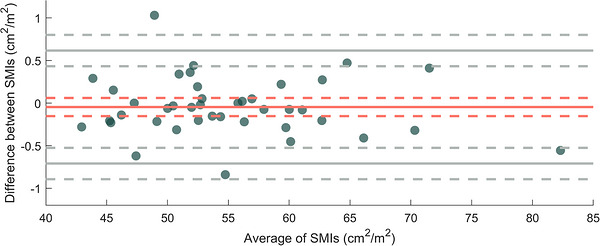
High concordance between the SMI measures obtained from the ImageJ‐method and the MIM‐method. Bland‐Altman diagram showing the agreement between the two methods (ImageJ‐method and MIM‐method) used to calculate the Skeletal Muscle Index (SMI). The x‐axis represents the average of the SMIs, the y‐axis shows the difference between the SMIs. The solid orange line at near zero indicates the mean difference (−0.04 cm^2^/m^2^) and the orange dashed lines indicate the 95% confidence interval for the mean difference. The solid grey lines show the limits of agreement (± 1.96 standard deviations from the mean difference, here ± 0.66 cm^2^/m^2^), demonstrating the range within which most differences are expected to fall. The dark green dots are individual data points showing variability across the measurements.

The concordance between SMI measures obtained with both methods, is shown on a Bland‐Altman plot (Figure 1). Ninety‐five percent of the values fell within the predefined range of agreement (± 0.66 cm^2^/m^2^), with a mean difference of −0.04 cm^2^/m^2^ between SMIs (*p* = 0.23). This demonstrates a very high level of agreement between the two methods, with no significant systematic bias nor random error.

To further assess clinical concordance, we compared the patients’ SP status based on the SMI obtained from each method. SP status was identical across all 41 patients. In other words, SP was consistently identified (or excluded) with both methods.

To quantify time efficiency, we measured the duration from CT image opening to final SMI calculation for both methods. The mean processing time was 3.5 ± 1 min using the MIM‐method and 10 ± 1 min using the ImageJ‐method (*p* < 0.001), indicating that the MIM‐method was nearly three times faster than the ImageJ‐method.

**FIGURE 2:**
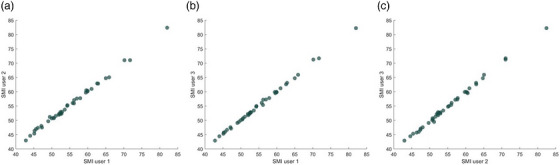
High correlation between SMI values obtained with the MIM‐method by three independent users. Three scatter plots comparing Skeletal Muscle Index (SMI) measurements taken by three different users. a. SMI values from User 1 are plotted against User 2, showing a strong positive correlation. b. SMI values from User 1 are compared with those of User 3, again indicating a high correlation. c. SMI values from User 2 are plotted against User 3, also showing a positive correlation.

To ensure that the MIM‐method results were not investigator‐dependent, we transmitted the MIM‐method step‐by‐step guide to two junior ROs and compared their SMI values among themselves and to those of the first investigator (Figure 2). The ICC was 0.99, indicating excellent reproducibility. We also compared how patients were labelled (sarcopenic or non‐sarcopenic) by each investigator and found a perfect agreement with a Fleiss’ k of 1. These results confirm the high inter‐investigator reliability of the MIM‐method.

Regarding procedural burden, qualitative interviews with the two junior ROs indicated that the method was “easy to understand”, “easy to learn”, “quick enough”, and imposed only a “small burden” beyond routine contouring.

**FIGURE 3:**
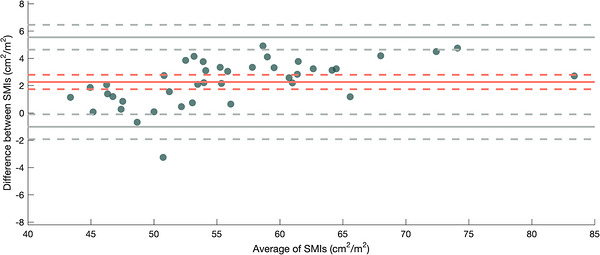
Systematic overestimation of SMI by the automated MIM‐workflow compared to the MIM‐ method. Bland‐Altman diagram showing the agreement between the two methods (Automated MIM‐workflow and MIM‐method) used to calculate the Skeletal Muscle Index (SMI). The x‐axis represents the average of the SMIs, the y‐axis shows the difference between the SMIs. The solid orange line at near two indicates the significant mean difference (2.3 cm^2^/m^2^) and the orange dashed lines indicate the 95% confidence interval for the mean difference. The solid grey lines show the limits of agreement (± 1.96 standard deviations from the mean difference, here ± 3.28 cm^2^/m^2^), demonstrating the range within which most differences are expected to fall. The dark green dots are individual data points showing variability across the measurements.

When comparing SMI values obtained with the semi‐manual MIM‐method versus the automated MIM workflow, we found a statistically significant mean difference of 2.3 cm^2^/m^2^ (*p* < 0.0001). The Bland‐Altman plot revealed a systematic overestimation of SMI by the workflow (fixed bias), which tended to increase with higher SMI values, indicating a mild proportional bias. Nevertheless, 95% of the data points fell within the predefined range of agreement (± 3.28 cm^2^/m^2^) (Figure 3).

The median processing time was 3.5 min (IQR 2.2–4.88) using the semi‐manual MIM‐method and 3.2 min (IQR: 1.8–4.52) using the automated MIM workflow (*p* < 0.001), confirming that automation further reduced the time required for SMI measurement.

## 5 DISCUSSION

Efficient methods to screen for SP in PCa patients are needed, given its high prevalence and impact on patient outcomes. Previous studies have shown that different software platforms used for CT‐based body composition analysis generally provide highly comparable measurements when standardized segmentation protocols are applied.^7^, ^8^, ^15^ Furthermore, recent developments in automated and AI‐based body composition analysis have reduced operator workload and facilitated large‐scale CT‐based SP assessment.^26^ Despite the growing evidence supporting the prognostic value of SP in oncology and significant advances in body‐composition assessment, routine SP screening remains limited in clinical practice.^27^ For radiation oncologists (ROs), who routinely use CT scans for PCa treatment planning, current approaches to assess SP may still be costly, time‐consuming, or challenging to integrate into existing workflows. Consequently, the main contribution of the present study is not the development of a new segmentation technique, but the demonstration that SP assessment can be reliably incorporated into a routine RT planning environment. We therefore evaluated the use of MIM®, a software platform already familiar to ROs, as a practical tool for SP assessment.

The MIM‐method proved reliable and reproducible for calculating SMI and identifying SP, showing strong agreement with the validated ImageJ‐method results. It also significantly reduced calculation time, improving efficiency nearly threefold. Furthermore, the MIM‐method was rated as easy and quick to perform, imposing only a small additional burden beyond standard contouring procedures. These findings are consistent with existing literature that supports CT‐based SP assessments^26^, ^28^ and suggest that MIM ® provides a practical and accessible approach for integrating SP screening into routine clinical practice.

The prevalence of SP in our cohort (22%) closely aligns with that reported in the recent meta‐analysis by Kovac et al. for patients with early PCa (32% [15.5%–48%]),^1^ suggesting that our sample may be representative of this population. However, the retrospective design, relatively small sample size, and single‐institution setting may limit the generalizability of the findings.

To measure muscle mass, we chose the L3 vertebral landmark because of its strong correlation with whole body muscle mass^29^, ^30^ and its systematic accessibility on CT simulations for PCa RT planning. Moreover, L3 measurements were used by Martin *et al.* ^6^ to define the specific SMI thresholds that were significantly associated with a low survival and that we used to define SP in this study. To account for differences in slice thickness between our CTs (2–3 mm) and those used by Martin *et al.* (5 mm), we chose to select two non‐adjacent axial slices rather than adjacent ones.

We demonstrated that further automation of the method within the MIM® platform is feasible and more time‐efficient. Nonetheless, Bland–Altman analysis revealed a systematic overestimation of SMI by the automated workflow, with a mild proportional bias, likely due to the differences in volume selection. In the semi‐manual MIM‐method, muscles were delineated on two slices to replicate Martin *et al*.’s methodology, whereas the automated MIM workflow performs segmentation across the entire L3 region. Although SMV is subsequently normalized by height to obtain SMA, using multiple slices may reduce the likelihood of missing peripheral muscle tissue, thereby resulting in slightly higher values. As well, the potential influence of slice selection in the semi‐manual MIM‐method on SMI measurements cannot be entirely ruled out. However, the excellent agreement with the reference method and the high inter‐observer reproducibility observed in this study suggest that its impact is likely limited.

This finding underscores the importance of interpreting SMI results with consideration of the specific segmentation methodology used and comparing them only with studies employing similar approaches. The SMI thresholds proposed by Martin *et al*.^6^ remain the most widely used across literature but may not be directly applicable to values derived from automated or volumetric measurements. Establishing method‐specific and population‐adjusted thresholds—ideally through receiver operating characteristic (ROC) curve analysis and area under the curve (AUC) evaluation—could improve the discrimination of SP status and help identify patients most likely to benefit from SP‐targeted interventions.

Although there is poor agreement among recent SP definitions proposed by the EWGSOP2 and the SP Definitions and Outcomes Consortium (SDOC),^31^ both include muscle strength assessment (e.g., using handgrip dynamometry^32^). We therefore encourage future studies to assess grip strength concomitantly with SMI to enable a more definitive clinical diagnostic of SP.

Importantly, our study demonstrates that the MIM‐method (with the step‐by‐step guide provided in this article in Appendix, Figure A1) already enables ROs to incorporate SP screening into their daily practice using tools available within their routine workflow. The automated workflow presented here provides a foundation for future optimization and may facilitate broader implementation without requiring additional software or training.

Future research should focus on validating these findings in larger, multi‐center trials to confirm the generalizability and clinical value of the MIM‐method across diverse institutional settings and patient populations. Moreover, adapting this approach for use with other widely adopted contouring platform (e.g., Eclipse®, RayStation®) could further enhance its accessibility and facilitate broader clinical implementation.

## 6 CONCLUSION

In summary, our study demonstrates that SP screening can be seamlessly integrated into RT planning using MIM® software, offering both accuracy and reproducibility while markedly reducing processing time. The development of an automated MIM‐workflow further enhances feasibility and time efficiency, although methodological refinement and validation of adapted SMI thresholds are still required to ensure consistency with established reference standards. This approach represents a crucial step toward a standardized, time‐efficient, and clinically accessible SP assessment in radiation oncology.

## AUTHOR CONTRIBUTIONS

**Pauline De Bruyn**: Conceptualization; data curation; investigation; methodology; project administration; writing—original draft; writing—review & editing. **Nicolas Coquelet**: Formal analysis; methodology; visualization; writing—original draft; writing—review & editing. **Paulo Ferreira**: Conceptualization; methodology; writing—original draft; writing—review & editing. **Madeline Michel**: Investigation; methodology; writing—review & editing. **Thomas Descamps**: Investigation; methodology; writing—review & editing. **Robbe Van den Begin**: Validation; writing—review & editing. **Dirk Van Gestel**: Supervision; validation; writing—review & editing. **Jean‐Charles Preiser**: Supervision; validation; writing—review & editing.

## CONFLICT OF INTEREST STATEMENT

The authors have no relevant conflicts of interest to disclose.

## ETHICS STATEMENT

The study was conducted in accordance with the Declaration of Helsinki, and approved by the Institutional Review Board (IRB) of the Institut Jules Bordet (protocol code CE3714, date of approval 12/10/2023).

## FUNDING INFORMATION

The authors have nothing to report.

## Supporting information



Supporting Information

## Data Availability

Research data are stored in an institutional repository and will be shared upon request to the corresponding author.

## Appendix

**TABLE A1:** SMI calculation formulas.

$\mathrm{SMA}\left(cm^{2}\right)=\frac{\mathrm{SMV}(cm^{3})}{\mathrm{Slice}\;\mathrm{thickness}(\mathrm{cm})}$
$\mathrm{Mean}\;\mathrm{SMA}=\frac{\left(\mathrm{SMA}\;\mathrm{slice}\;1+\mathrm{SMA}\;\mathrm{slice}\;2\right)}{2}$
$\mathrm{SMI}\;\left(cm^{2}/m^{2}\right)=\frac{\mathrm{Mean}\;\mathrm{SMA}}{\mathrm{Body}\;\mathrm{surface}\;(\mathrm{Heigh}t^{2})}$

SMV, Skeletal Muscle Volume; SMA, Skeletal Muscle Area; SMI, Skeletal Muscle Index.

Figure A1. Full step‐by‐step guide and Excell of the MIM‐method.

Figure A2. Illustration of the full MIM‐method validation's study design.
