# Supplementary material for: Integration of sarcopenia screening into radiotherapy planning: Validation of a time‐efficient SMI measurement method using MIM software in prostate cancer
Source: J Appl Clin Med Phys. 2026 Jul 9;27(7):e70694. doi: 10.1002/acm2.70694 (PMC13351315; doi:10.1002/acm2.70694)
Supplement: Supplementary file 1 — Supporting Information [file ACM2-27-e70694-s001.pdf]

Guide to recreate the workflow '"SARCOPEMIM" with MIM's Workflow Builder

1. Toggle Contours
    a. All the contours
    b. Series : CT
    c. Hide contour

2. Suspend workflow with wanted message : « In the next step of the workflow, you will be prompted to identify the height of L3. You should zoom in and prepare your window in order to facilitate the contour. »

3. Create contour
    a. Series : CT
    b. Output name : L3

4. Create Dynamic Stat
    a. Stat source : L3
    b. Stat type : volume (ml, pixel^3)
    c. Natural value
    d. Output : Dyna Stat 1

5. Reduce Dynamic Stat
    a. Dynamic Stat : Dyna Stat1
    b. Series : CT
    c. Method : return statistic for the first contour in the list
    d. Lower bound
    e. Upper bound
    f. Skip Nans
    g. Outputs : volume ; Stat2

6. Compare Data
    a. Compare Number
    b. Volume
    c. Equal to
    d. Number (0)
    e. Output : Empty

7. Using running condition « empty » : Set Series to Contour
    a. Series : CT

8. Using running condition « empty » : Set Active Contour
    a. Contour : L3

9. Using running condition « empty » : Activate Tool
    a. Tool : 3D Brush

10. Using running condition « empty » : Suspend workflow with the message : « In the next step of the workflow, you will be prompted to identify the height of L3. You should zoom in and prepare your window in order to facilitate the contour. »

11. Hide Processing : Hide Processing
12. Contour Settings
    a. Contour : L3
    b. Color : Cyan
    c. Settings : Lower lock : 150

13. Clean
    a. Contour : L3
    b. Method for cleaning : clean volumetrically
    c. Clean regions beyond threshold
    d. Threshold : 10
    e. Current frame

14. Expand/Contract
    a. Contour : L3
    b. Destination : L3
    c. Parameters:
        i. Operation : Expand
        ii. Use same distance for all directions : true
        iii. Distance (all directions) : 0.3 cm
        iv. Do not overlap with hard edges : false
    d. Output : L3

15. Fill Holes
    a. Contour : L3
    b. Method : fill volumetrically

16. Clean
    a. Contour : L3
    b. Method : clean each axial slice
    c. Current frame

17. Hide processing : show processing

To introduce height and weight values in order to obtain the BMI automatically and be able to export it out of the workflow into the designed excel file:

18. Enter Number
    a. Text : Introduce the height of the patient (m) in the box below
    b. Default Value
    c. Output : Height

19. Enter Number
    a. Text : Introduce the weight of the patient (kg) in the box below
    b. Default Value

c. Output : weight

20. Stat Formula
    a. Parameters :
        i. Formula : Weight / (Height * Height)
        ii. Output Statistic : BMI
        iii. Output : BMI
    b. Output : BMI
21. Find Contours*
    a. Search Series
    b. Series: CT
    c. Search All Contours
    d. Text: Bag_bowel
    e. Simple Search
    f. Contour Interpreted Type: None
    g. Volumetric Only
    h. Output: Bag_Bowel

22. Run Condition; Invert; Run condition: Bag_Bowel**
    a. Find Contours
        i. Search Series
        ii. Series: CT
        iii. Search All Contours
        iv. Text: Bowel
        v. Simple Search
        vi. Contour Interpreted Type: None
        vii. Volumetric Only
        viii. Output: Bag_Bowel

23. Run Condition; Invert; Run condition: Bag_Bowel
    a. Find Contours
        i. Search Series
        ii. Series: CT
        iii. Search All Contours
        iv. Text: Bowel_Bag
        v. Simple Search
        vi. Contour Interpreted Type: None
        vii. Volumetric Only
        viii. Output: Bag_Bowel

24. Find Contours
    a. Search Series
    b. Series: CT
    c. Search All Contours
    d. Text: .M_Bone
    e. Simple Search
    f. Contour Interpreted Type: None

g. Volumetric Only
h. Output: Bone

25. Run Condition; Invert; Run condition: Bone
    a. Find Contours
        i. Search Series
        ii. Series: CT
        iii. Search All Contours
        iv. Text: Sacrum
        v. Simple Search
        vi. Contour Interpreted Type: None
        vii. Volumetric Only
        viii. Output: Bone

26. Run Condition; Invert; Run condition: Bone
    a. Find Contours
        i. Search Series
        ii. Series: CT
        iii. Search All Contours
        iv. Text: Bones
        v. Simple Search
        vi. Contour Interpreted Type: None
        vii. Volumetric Only
        viii. Output: Bone
27. Create contour
    a. Series : CT
    b. Output name : SMV

28. Create contour
    a. Series : CT
    b. Output name : Delete

29. Boolean Operations
    a. Calculation: (Body NOT) OR Skin OR Bowel_Bag OR Bone
    b. Destination: Delete
    c. All frames
    d. Output: Delete

30. Boolean Operations
    a. Calculation: SMV NOT
    b. Destination: SMV
    c. All frames
    d. Output: SMV

31. Contour Settings
    a. Contour: SMV
    b. Settings:
        i. Lower Lock: - 29

ii. Upper Lock: 150

32. Boolean Operations
    a. Calculation: SMV ‘subtracts the contour to the right from the contour to the left’ Delete
    b. Destination: SMV
    c. All frames
    d. Output: SMV

33. Boolean Operations
    a. Calculation: L3 ‘ Expands or contracts the size of the contour’
        i. Settings
            1. Set all directions the same: 30 cm
    b. Destination: unmapped
    c. All frames
    d. Output: Volume

34. Boolean Operations
    a. Calculation: SMVAND Volume
    b. Destination: SMV
    c. All frames
    d. Output: SMV

To clean the created SMV volume and facilitate the evaluation and adaptation of this volume

35. Toggle Contours
    a. All the contours
    b. The list of contours to use: CT
    c. Hide Contour

36. Toggle Contours
    a. A specified list of contours
    b. The list of contours to use: SMV
    c. Show contour

37. Clean
    a. Contour : SMV
    b. Method : Clean volumetrically
        i. Clean regions below threshold
    c. Threshold 0.2
    d. All frames

38. Smooth
    a. Contour: SMV

39. Set active contour
    a. Contour: SMV

40. Active Tool
    a. Localize

41. Suspend Workflow
    a. Text: *Take a moment to review the contoured volume. Refine it with the eraser tool until only muscular tissue remains — removing any great vessels, bowel, kidneys, spinal canal, nerve roots and intervertebral disc from the contour.*

42. Find Contour Statistic
    a. Contour: SMV
    b. Return Statistic for the list of contour in the list
    c. Exclude Empty Contours
    d. Volume (ml, pixel^3)
    e. Output: VolumeSMV

43. Launch Extension
    a. Longitudinal dimenson~MIM***

44. Find Contour Statistic
    a. Contour: L3
    b. Return Statistic for the first contour in the list
    c. Exclude Empty Contours
    d. Statistic from Extension
        i. Statistic Key: long_lenght
        ii. Namespace: MIMEX

45. Look Up Dicom
    a. Series: CT
    b. Tag:
        i. Search by tag name or number
        ii. PatientID
    c. Number
    d. Integer String (IS)
    e. Exactly one
    f. Output: ID

46. Stat Formula
    a. Parameters:
        i. Formula: HeightSMV*01
        ii. Color Provider: HeightSMV
        iii. Output Statistic: HeightSMV
    b. Output: HeightSMV

47. Stat Formula
    a. Parameters:
        i. Formula: VolumeSMV/HeightSMV
        ii. Color Provider: HeightSMV

        iii. Output Statistics: Area
    b. Output: Area

48. Stat Formula
    a. Parameters:
        i. Formula: Height * Height
        ii. Color Provider: Height
        iii. Output Statistics: Body_Surface
    b. Output: Body_Surface

49. Stat Formula
    a. Parameters:
        i. Formula: Area/Body_Surface
        ii. Color Provider: Area
        iii. Output Statistics: SMI
    b. Output: SMI

50. Suspend workflow with wanted text “The data has been calculated and it's ready to be exported to the destined excel file. Make sure the excel file is not already open.’’

51. Write to CSV
    a. Include directory of the file
    b. Append
    c. Line:
        i. Element
            1. Text: ID
            2. Field Value: ID
        ii. Element
            1. Text: Height_m
            2. Field Value: Height
        iii. Element
            1. Text: Weight_kg
            2. Field Value: Wight
        iv. Element
            1. Text: BMI
            2. Field Value: BMI
        v. Element
            1. Text: Volume_SMV
            2. Field Value: VolumeSMV
        vi. Element
            1. Text: Height_L3_cm
            2. Field Value: HeightSMV
        vii. Element
            1. Text: Area_cm2
            2. Field Value: Area
        viii. Element
            1. Text: Body_Surface_m2
            2. Field Value: Body_Surface

ix. Element
1. Text: SMI
2. Field Value: SMI

52. Save Session
a. User Session
b. Text: SARCOPEMIM
c. Associated Series: Unmapped
d. An optional workflow: unmapped
e. Output: SARCOPEMIM

The names of the structures created during the workflow are completely customizable. They can and should be adapted to what suits best the practice of the clinic implementing this workflow.

*Search for existing contours that can be subtracted from the previously created SMV volume (defined at the level of L3). Please note that contour names vary by institution. For example, in our workflow, bowel-related structures are labeled as *"bag_bowel"*, *"bowel"*, etc. Therefore, the search terms in this step must be adapted to the naming conventions used at the implementing institution.

**Step 22, in particular, should be repeated as many times as necessary to ensure that all relevant bowel contours are identified. In our case, the search includes *"bowel"* and *"bowel_bag"*, as these are the only variants corresponding to what other institutions may call *"bag_bowel"*. A similar procedure was applied to both bowel and bone contours.

***This is an extension provided by MIM that makes possible the extraction of the height of a contour.
